# Molecules to Ecosystems: Actinomycete Natural Products *In situ*

**DOI:** 10.3389/fmicb.2016.02149

**Published:** 2017-01-17

**Authors:** Scott W. Behie, Bailey Bonet, Vineetha M. Zacharia, Dylan J. McClung, Matthew F. Traxler

**Affiliations:** Department of Plant and Microbial Biology, University of California, Berkeley, BerkeleyCA, USA

**Keywords:** actinomycetes, natural products, *in situ*, chemical ecology, microbial interactions

## Abstract

Actinomycetes, filamentous actinobacteria found in numerous ecosystems around the globe, produce a wide range of clinically useful natural products (NP). In natural environments, actinomycetes live in dynamic communities where environmental cues and ecological interactions likely influence NP biosynthesis. Our current understating of these cues, and the ecological roles of NP, is in its infancy. We postulate that understanding the ecological context in which actinomycete metabolites are made is fundamental to advancing the discovery of novel NP. In this review we explore the ecological relevance of actinomycetes and their secondary metabolites from varying ecosystems, and suggest that investigating the ecology of actinomycete interactions warrants particular attention with respect to metabolite discovery. Furthermore, we focus on the chemical ecology and *in situ* analysis of actinomycete NP and consider the implications for NP biosynthesis at ecosystem scales.

## Introduction

Of the top ten leading causes of death in 1900, three were bacterial infections, and they accounted for greater than one third of total deaths in the US (Jones et al., 2012). Today, bacterial infections are absent from the top ten list due to the discovery and development of antibiotics from fungi and bacteria. In 1943 the first actinomycete-produced therapeutic, streptomycin, was discovered by Albert Schatz, Elizabeth Bugie, and Selman Waksman and was rapidly put into use to treat tuberculosis (Schatz et al., 1944). Actinomycetes are remarkable Gram-positive, filamentous, bacteria responsible for producing an estimated 70% of the antibiotics used in human therapy (Bérdy, 2005), making them the most robust natural source of antibiotics. Antibiotics and other unique compounds produced by actinomycetes are known as natural products (NP), or secondary or specialized metabolites (SM).

Historically, most discoveries of NP from actinomycetes have involved their growth in rich media, in monoculture, which is strikingly different from their natural environment. During the “golden era” of antibiotics discovery in the 1950s and 1960s, this method worked remarkably well, and lead to a rapid increase in the number of medically relevant NP found from bacteria, including anti-cancer therapeutics, immunosuppressants, and anthelminthics (Bérdy, 2005). From the 1980s and onward the discovery of novel NP from actinomycetes began dropping precipitously as compound re-discovery emerged as a major impediment (Van Middlesworth and Cannell, 1998). The 1990s and 2000s witnessed the large-scale shutdown of most commercial efforts to isolate novel compounds from actinomycetes. This situation was, and continues to be, exacerbated by the rise of antibiotic resistance in pathogenic microbes.

Now, in the post-genomic era, with thousands of actinomycete genome sequences available, we have come the realization that actinomycetes possess the genetic capability to produce a multitude of NP never before observed in the laboratory. This realization, coupled with advances in genetic tools, has rekindled interest in exploring actinomycete NP in new and creative ways. Some of these strategies include physiological or genetic manipulation, sampling actinomycetes from new sources, using methods to isolate rare actinomycetes, and co-culturing (Bode et al., 2002; Traxler et al., 2013; Yamanaka et al., 2014; Harvey et al., 2015; Pimentel-Elardo et al., 2015). Beyond these, efforts that capitalize on genome mining supported by massive sequencing efforts (Doroghazi et al., 2014; Ju et al., 2015; Goering et al., 2016), high-throughput heterologous expression of NP gene clusters from environmental DNA (Subramani and Aalbersberg, 2013; Milshteyn et al., 2014; Iqbal et al., 2016), NP pathway re-factoring and heterologous expression, *in situ* cultivation of ‘unculturable’ bacterial strains [e.g., the iChip, (Ling et al., 2015)], and the MS-guided genome-mining (termed peptidogenomics) (Kersten et al., 2011), have been undertaken. Many of these efforts have yielded exciting payoffs in terms of compound discoveries in just the past few years, hinting that a new era of discovery may be at hand.

While developing new ways to discover NP has been, and will certainly continue to be, an important goal, our understanding of the ecological role of these NP is relatively primitive. We suggest that understanding the ecological relevance of these molecules for the microbes that make them is of great importance to inform future strategies for NP discovery. For example, if we can fully understand the environmental cues that stimulate NP production *in situ* we can exploit these cues in the laboratory to induce normally silent NP gene clusters. Our nascent understanding has lead researchers to propose that NP may play a role in competition for resources, communication and signaling with other organisms, or defense of a symbiotic partner (Davies et al., 2006; Linares et al., 2006; Ryan and Dow, 2008; Cornforth and Foster, 2013; Abrudan et al., 2015). While the initial experiments underpinning these hypotheses are important first steps toward understanding NP ecology, relatively few studies have actually detected the effects of NP *in situ*.

This review focuses on studies where actinomycete NP biosynthesis has been shown *in situ*. We briefly review these systems and consider the possible implications of NP activity within their respective ecological contexts, both at a proximate and indirect level. Actinomycete NP from soil or marine sediments have been addressed in recent reviews (Subramani and Aalbersberg, 2012; Traxler and Kolter, 2015), and therefore we do not address them here. The role of actinomycetes in symbiotic relationships has also been recently reviewed, and thus we emphasize studies post-2012 (Seipke et al., 2012). We conclude by considering new tools and experimental paradigms that hold promise for exploring the chemical ecology of actinomycete NP in the future.

## Use of Actinomycete Natural Products By Fungus-Farming Ants

**FIGURE 1:**
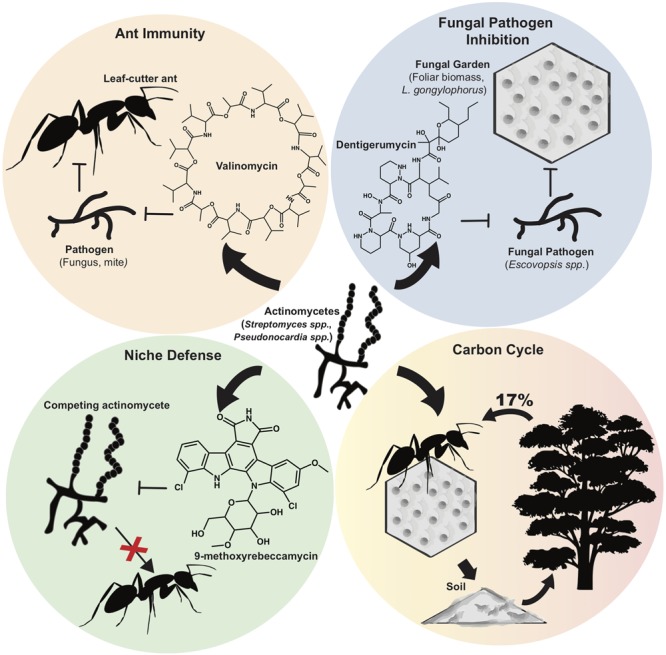
**Roles of actinomycete natural products in the leaf-cutter ant ecosystem.** Different actinomycete species isolated from attine ant integument (*Streptomyces* spp., *Pseudonocardia* spp., etc.) secrete natural products with various functions and roles. (*Top left*) *Streptomyces* spp. confer ant immunity by producing valinomycin, antimycins, and actinomycins, which inhibit pathogens and parasites. (*Top Right*) Actinomycetes produce a range of antifungals including dentigerumycins, candicidin, and nystatin variants, which inhibit fungal pathogens (e.g., *Escovopsis* spp.) but are not detrimental to the attine ant fungal symbiont, *L. gongylophorus*. (*Bottom Left*) *Pseudonocardia* competing for residency on the ant integument use plasmid-encoded niche defense mechanisms by synthesizing 9-methoxyrebeccamycin. (*Bottom Right*) Natural products from actinomycetes help preserve the attine ant community, which in turn, affects the larger ecosystem. Leaf-cutter ants forage up to 17% of the foliar biomass and the *L. gongylophorus* symbiont helps degrade organic material, which collectively contributes to carbon turnover.

One of the most extensively characterized natural systems that harbor actinomycetes is that of the leaf-cutter ants. Actinobacteria associated with fungus-growing leaf-cutter ants produce NP that have been shown to have key roles in shaping this ecosystem (**Figure 1**) (Currie et al., 1999). Primarily found in the Americas, leaf-cutter ants (including the genera *Atta* and *Acromyrmex*) maintain a delicately balanced network that includes their fungal food source, *Leucoagaricus gongylophorus*, and actinomycetes, which produce antimicrobials to protect the fungal gardens against pathogen invasion (Schultz and Brady, 2008; Hölldobler and Wilson, 2010; Andersen et al., 2015). If the balance of the microbial community is perturbed, fungal pathogens belonging to the *Escovopsis* genus can invade the fungal garden, resulting in reduced garden biomass and the eventual destruction of entire ant colonies (Currie et al., 2003; Reynolds and Currie, 2004). Ultimately, the NP synthesized by the symbiotic actinobacteria play a critical role in preserving the overall fitness of leaf-cutter ant colonies by protecting *L. gongylophorus* from pathogen infection.

Actinomycetes predominantly belonging to the *Streptomyces* and *Pseudonocardia* genera are also directly associated with the cuticle of leaf-cutter ants. These actinomycetes were studied in assays against invading fungal species and were found to produce a suite of antifungals including antimycins, candicidin, dentigerumycin, and nystatin variants which suppress *Escovopsis* and other potential pathogens while leaving *L. gongylophorus* unharmed (Haeder et al., 2009; Seipke et al., 2012; Dângelo et al., 2016). More recently, Sit et al. (2015) discovered, and chemically characterized, new antifungals related to dentigerumycin, called gerumycins, synthesized by *Pseudonocardia* spp. associated with both *Apterostigma dentigerum*, and the *Trachymyrmex cornetzi* ant (Sit et al., 2015). To add another level of complexity to this ecological system, the NP synthesized by leaf-cutter ant actinomycetes may also serve to fend off closely related bacteria that may displace the resident strain (Poulsen et al., 2007). This antagonism between different species of ant-associated *Pseudonocardia* was pronounced in more distantly related species, suggesting that competition between *Pseudonocardia* have shaped this association from its evolutionary origin (Poulsen et al., 2007). Consistent with this idea, Van Arnam et al. (2015) recently found that one *Pseudonocardia* strain produced a novel rebeccamycin analog that inhibited the growth of competing *Pseudonocardia* (**Figure 1**). PacBio sequencing of these strains revealed variations in the rebeccamycin BGCs which were located on plasmids, suggesting plasmid-encoded niche defense (Van Arnam et al., 2015). Furthermore, rebeccamycins are of interest to human health since they possess anti-tumor activities and are currently being tested in clinical trials (Xu and Her, 2015).

Interestingly, recent evidence suggests that the direct interaction of actinomycetes with their leaf-cutter ant hosts is beneficial for worker ant protection from pathogenic fungi and bacteria (Schoenian et al., 2011; Mattoso et al., 2012; de Souza et al., 2013). With high resolution mass spectrometry (LC-ESI-HR-MS), and matrix-assisted laser desorption ionization (MALDI) imaging on the bodies of the ants, Schoenian et al. (2011) identified valinomycins, actinomycins, and antimycins from *Streptomyces* isolates associated with the integument of *Acromyrmex echinatior* workers. In their bioassays, Schoenian et al. (2011) demonstrated the inhibitory effects of these NP on insect pathogens such as *Metarhizium anisopliae*, and *Cordyceps militaris*, and on *Escovopsis weberi* and *Fusarium decemcellulare*, which are fungal pathogens of the fungus garden. Strikingly, the MALDI-imaging data clearly showed the localization of valinomycin secreted by *Streptomyces* on the ant cuticle at varying concentrations. Taken together, these data suggest that the presence of integumental biofilms and their secreted NP work in concert to protect fungal gardens from invading bacteria and fungi, while simultaneously contributing to worker ant immunity by inhibiting the growth of entomopathogens (**Figure 1**) (Schoenian et al., 2011). To corroborate the importance of actinomycete NP in their protective roles against ant-specific pathogens, cuticle-associated biofilms were removed with antibiotic treatment, thereby exposing the ants to attack by the entomopathogenic fungus *M. anisopliae*, which resulted in increased mortality of *Acromyrmex* ants (Mattoso et al., 2012). Furthermore, the immunity of *Acromyrmex subterraneus* ants with and without their symbiotic actinomycetes was evaluated. In their study, de Souza et al. (2013) observed that young worker ants lose their bacterial coating and mature into older, external worker ants. Furthermore, they found that in the absence of their associated bacteria, the young ants had an altered innate immune response, indicating that the actinomycetes confer protection to young internal workers until their immune systems have matured (de Souza et al., 2013). These works collectively stand as a rare concrete example in which the actinomycetes have been removed from the system and their influence ascertained, with the overall result being that their hosts became vulnerable to disease. The authors of these works logically conclude that the most likely reason for this is the activity of the NP made by the actinomycetes *in situ*. Ultimately this hypothesis might be tested in the future by re-colonizing ants with mutant actinomycete strains lacking the ability to make specific NP. The study of Seipke et al. (2011) takes a step toward conducting this type of experiment as they generated *Streptomyces* mutant strains unable to synthesize candicidin and antimycin (verified by PCR and LC-MS) and tested its bioactivity toward fungi *in vitro* (Seipke et al., 2011). The results, however, were inconclusive in terms of mutant effect, therefore it was not tested *in situ*. This highlights the challenge of executing complex, *in situ* microbial ecology.

**FIGURE 2:**
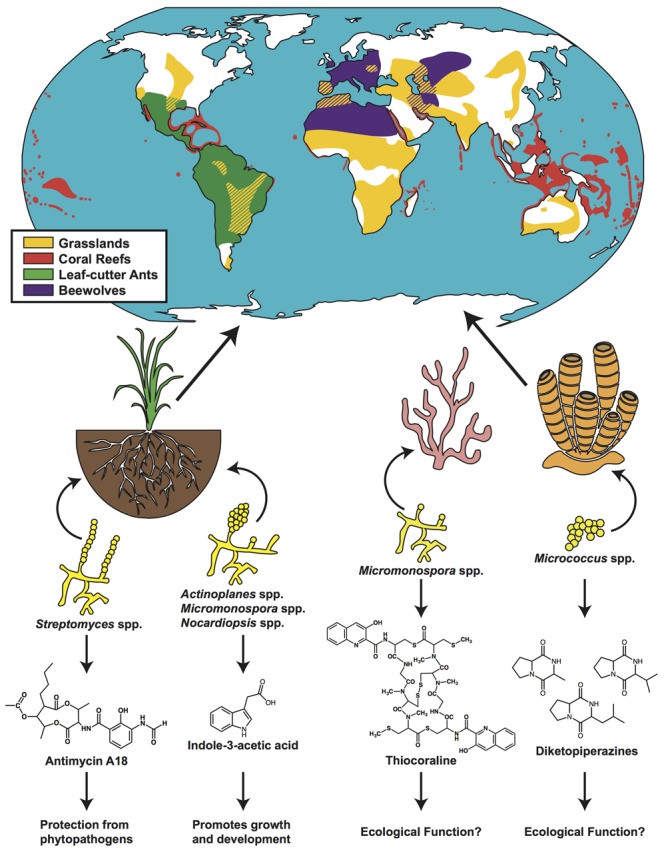
**Actinomycete-produced natural products aid in ecosystem function across the globe.** Distributions of grasslands (yellow), coral reefs (red), leaf-cutter ants (green), and beewolves (purple) are denoted on a map of the globe. Representative actinomycetes and the natural products that they produce in rhizosphere, coral and sponge communities are shown. Distribution maps adapted from: www.thinglink.com/scene/655045986153922561 (grasslands); www.antweb.org/antblog/2011/03/geographic-range-of-leaf-cutter-ants-don-indianapolis-in-usa.html (leaf-cutter ants); www.grida.no/graphicslib/detail/distribution-of-coldwater-and-tropical-coral-reefs_1153 (coral reefs); Herzner and Strohm, 2008 (beewolf).

As they are the dominant herbivores in much of the Neotropics (Costa et al., 2008), the foraging activity of leaf-cutter ants has a large impact in the overall function of their ecosystems (**Figures 1** and **2**). These ants forage up to 17% of the foliar biomass and contribute substantially to carbon turnover in their forest habitats (Costa et al., 2008). Their fungal crop, *L. gongylophorus*, produces lignocellulases capable of breaking down plant polymers (Aylward et al., 2013), which contribute significantly to organic material degradation and re-introduction into the neotropical terrestrial environment. As described above, actinomycete NP likely play a role in protecting the ant’s fungal gardens from invasion, and also play a role in protecting the ants themselves from parasites and diseases. While equivocally assessing the direct contribution of these NP is extremely challenging at an ecosystem scale, this body of evidence supports the notion that actinomycete NP are intimately involved in maintaining one of the largest herbivory cycles on earth.

We also note that actinomycetes are associated with non-attine ants such as the Japanese carpenter ant (*Camponotus japonicus).* The actinomycetes *Nocardia camponoti* sp. and *Promicromonospora alba* sp. were recently isolated from the heads of these ants, and their genomes were sequenced (Guo et al., 2016; Liu et al., 2016). While the NP repertoire of these strains has not yet been explored, further study of these newly reported interactions might lead to the discovery of novel bioactive compounds that play a role in shaping the Japanese carpenter ant’s ecosystem.

## Offspring Protection Via Actinomycete Natural Products By Beewolf Wasps

The beewolf digger wasps (*Philanthus* spp., Hymenoptera, Crabronidae) live in a symbiosis with the streptomycete *Candidatus Streptomyces philanthi*, which is housed within the antennal glands of the female beewolves (Kaltenpoth et al., 2005). Female wasps deposit this bacterial symbiont onto the inner walls of the protective underground burrows where they lay their eggs. These streptomycetes are incorporated into the silk as the larvae begin spinning their cocoons (Kaltenpoth et al., 2006). The cocoons remain immobile in the humid burrows, and are thus easy targets for fungal and bacterial pathogens that live in the soil or are introduced by the honeybees brought into the burrow as a food source for the larvae (Kaltenpoth et al., 2005). However, the streptomycete symbionts protect the cocoons by secreting nine antimicrobial or antifungal compounds onto the outer surface of the cocoon (Kroiss et al., 2010; Seipke et al., 2012; Koehler et al., 2013).

Kroiss et al. (2010) were able to detect three of these compounds, the antibiotics piericidin *A*_1_, piericidin *B*_1_, and streptochlorin *in situ*. They further elucidated that there was a uniform distribution of all three compounds on the outer surface of the cocoon as compared to the inner surface (Kroiss et al., 2010). This distribution of antimicrobials suggests that the metabolites protect the cocoon from invading pathogens on the outside, but are at lower concentrations inside to prevent disruption of larval development. Four of the nine antimicrobials were shown to be inhibitory to the growth of 10 soil fungi and bacteria that would likely pose a threat of infection to the cocoons such as *Aspergillus* and *Metarhizium* (Strohm and Linsenmair, 2001; Kroiss et al., 2010). *In situ* analysis with MALDI-TOF/MS in conjunction with fluorescence *in situ* hybridization (FISH), showed that the insect associated streptomycetes co-localize with piericidin *A*_1_ and *B*_1_ (Kaltenpoth et al., 2016). This data illustrates the role of *Streptomyces* produced antimicrobials *in situ* that protect the beewolf cocoon from potential microbial infections, ensuring offspring survival.

At an ecosystem level, it is challenging to imagine what the overall effect of these actinomycete-produced compounds might be. However, this system is notable because it is one of the few examples in which NP from actinomycetes have been visualized *in situ*. To the extent that this system may be manipulated at the genetic and chemical level, it may represent one of the best opportunities for assessing the direct impact, and therefore the *bona fide* role, of a NP made by an actinomycete in a natural setting.

## Actinomycete Specialized Metabolism in the Rhizosphere

Actinomycetes that inhabit the rhizosphere have been shown to secrete a wide range of NP that can contribute to defense against pathogenic bacteria and fungi, and aid in plant nutrient scavenging (El-Tarabily et al., 2009; Solans et al., 2011; Rungin et al., 2012; Harikrishnan et al., 2014). Rhizosphere soils contain relatively high levels of actinobacteria (Hirsch and Mauchline, 2012), and the relationship between soil actinomycetes and plant roots can have dramatic effects on plant health.

Root-colonizing *Streptomyces* have been found to produce a number of antifungal and antimicrobial compounds *in situ* such as staurosporine, 3-acetonylidene-7-prenylindolin-2-one, diastaphenazine, and antimycin A18 (Yan et al., 2010; Li et al., 2014, 2015; Zhang et al., 2014). These *in situ* analyses offer a critical lens for beginning to understand bioactive compound production in the natural context of the rhizosphere. Studies examining the large-scale effect of rhizosphere actinomycetes have been done with respect to plant health and disease resistance. For example, when cucumber plants (*Cucumis sativus*) were inoculated with one or a combination of three endophytic actinomycetes, *Actinoplanes campanulatus, Micromonospora chalcea*, or *Streptomyces spiralis*, the overall effects of *Pythium aphanidermatum*, a soil-borne, fungal phytopathogen, were mitigated. Specifically, host plants showed significantly less root and crown rot, and were healthier overall (El-Tarabily et al., 2009). A number of other studies have shown similar results, in various plant systems, where root-inoculated actinomycetes were able to protect the plant from harmful pathogen invasion (Xue et al., 2013; Nabti et al., 2014; Solans et al., 2016). In a notable example, *Streptomyces lividans* was shown to produce prodiginines on the roots of *Arabidopsis thaliana*, which antagonized the particularly damaging fungal pathogen *Verticillium dahlia* (Meschke et al., 2012). The ability of rhizosphere-inhabiting actinomycetes to produce a large range of metabolites antagonistic to phytopathogens, and subsequently provide protection, point to an important role for soil actinomycetes in ensuring plant health and primary production (**Figure 2**).

Soil actinomycetes are also known to enhance plant health by stimulating plant growth and development through the production of phytohormones (Solans et al., 2011; Harikrishnan et al., 2014). Endophytic actinomycetes such as *Nocardiopsis, Actinoplanes* spp., and *Micromonospora* have been found to produce a number of important phytohormones such as indole-3-acetic acid (IAA) (**Figure 2**) and indole-3-pyruvic acid (IPYA), compounds required for fundamental plant functions including coordinated cell growth, and gene regulation (Subramaniam et al., 2016). This suite of compounds has been specifically shown to promote growth and development in wheat, lettuce, rye, and tomato (Merzaeva and Shirokikh, 2010; Bonaldi et al., 2015; Abbamondi et al., 2016; Toumatia et al., 2016). Directly delineating microbially produced phytohormones, as opposed to phytohormones made by the host, is technically challenging. However, the *in vitro* production of these compounds by root colonizing, or rhizosphere competent, actinomycetes suggests a potential means by which the soil microbiome is able to impact plant health (**Figure 2**).

Soil *Streptomyces* are also capable of enhancing the overall nutrient acquisition efficiency of plants by promoting the growth of critical symbiotic nitrogen fixers such as Rhizobia, and by aiding in root nutrient scavenging. Leguminous plants enter into a symbiosis with bacteria of the *Rhizobiales* clade in which the bacterium stimulates the plant to produce a root nodule that house the bacteria. In turn the bacteria fix atmospheric nitrogen, making it available to the plant. When chickpea plants were inoculated with *Streptomyces*, the result was increased nodulation as well as an increase in nodule size suggesting an overall stimulating effect on the nitrogen-fixing capability of the plant (Gopalakrishnan et al., 2015). This is especially notable given that fixed nitrogen is a limiting resource for plants in all systems. The production of siderophores by actinomycetes within the rhizosphere, or directly on the root, can specifically aid the plant in scavenging iron from surrounding soils (Rungin et al., 2012). Through the production of their own siderophores, plants are able to scavenge the soil for soluble iron (Ma, 2005). However, plants are also able to uptake iron-bound, actinomycete-produced, siderophores, increasing their overall iron uptake efficiency.

The example studies highlighted above point to a ubiquitous association between actinomycetes and plant roots in the rhizosphere. By in large, this association is beneficial for plants as they may receive multiple chemical inputs from actinobacteria, including siderophores, phytohormones, antibiotics, and antifungals that inhibit potential pathogens (Solans et al., 2011; Rungin et al., 2012; Harikrishnan et al., 2014; Li et al., 2014; Zhang et al., 2014). Most studies have examined actinomycete/plant relationships in the context of food crops, some of which are grasses such as wheat, sorghum, rye, maize, and rice (Bressan, 2003; Merzaeva and Shirokikh, 2010; Harikrishnan et al., 2014; Toumatia et al., 2016). Beyond this, metagenomic and culture-based studies of rhizosphere soil from grasslands have revealed a wide array of plant-associated actinomycetes (Lugo et al., 2007; Delmont et al., 2012). Based on this collective evidence, we suggest that chemical interactions between actinomycetes and plants (especially non-woody plants) are likely to influence plant health at an ecosystem scale, especially in grasslands (**Figure 2**). Though the exact *in situ* role of each actinomycete produced NP is not fully understood, it is clear that the presence of actinomycetes alter the plant’s susceptibility to pathogens, nutrient uptake, and growth. Of course, other microbes also likely to play a major role in these complex interaction webs, but the large number of NP produced by actinomycetes may make their role unique.

## Actinomycete Natural Products in Marine Organisms

The ocean covers 70% of the earth’s surface and is responsible for 50% of the earth’s daily primary productivity, which amounts to an estimated 140 million tons of carbon production per day (Field et al., 1998). Actinomycetes have been found in many marine habitats including marine sediments, estuaries, fish, mollusks, mangroves, and seaweeds, and marine actinomycetes have long been a productive source of prospecting for novel NP. Isolating actinomycetes from marine sediments has proven to be a successful strategy for discovery of potent bioactive molecules (Fenical et al., 2009) and coupling this with sequencing efforts has shown that there is great potential still to be discovered (Udwary et al., 2007; Ziemert et al., 2014). For now we will focus on coral reefs and marine sponges as these are the best studied marine actinomycete environments. Studies have measured the diversity and abundance of actinobacterial symbionts, finding that they are ubiquitously and stably associated within these systems (Abdelmohsen et al., 2014; Braña et al., 2015; Mahmoud and Kalendar, 2016). Subsequently, actinomycetes have been isolated from various sponges and corals to investigate their antimicrobial activities and potential to produce novel bioactive compounds (Schneemann et al., 2010; Cheng et al., 2015; Kuang et al., 2015; Pham et al., 2015; Mahmoud and Kalendar, 2016). The isolates have proved to be a rich source of NP with antifungal, antimicrobial, anti-cancer, and anti-HIV compounds (Abdelmohsen et al., 2014).

The majority of these studies involved isolating the actinomycetes and culturing them in the laboratory setting. Comprehensive laboratory analyses done on sponge associated actinomycetes has found that a number of different species are culturable from marine sponges, and that they are capable of producing bioactive secondary metabolites (Schneemann et al., 2010; Xi et al., 2012). With respect to the sponge *Halichondria panicea*, for example, five actinomycete genera were isolated and the bacterial extracts were found to contain secondary metabolites with bioactivities (Schneemann et al., 2010). However, ecological analysis of marine environments remains challenging, and there are few studies that have reported *in situ* data. Recently, however, Yarnold et al. (2012) used MALDI-MS imaging to map the spatial distribution of brominated pyrrole-2-aminoimidazole alkaloids, which have cytotoxic and anti-fouling properties, on cross sections of the marine sponge *Stylissa flabellata*. These compounds were found in discrete locations within the sponges and were present in high amounts near sites most susceptible to predation and biofouling. There is yet no clear delineation between the suite of metabolites produced by the sponge, and those produced by associated microorganisms, but gradients and pockets of metabolite production suggest that these are likely to be microbially produced. In one specific example, three diketopiperazines, one of which has antibiotic properties, that were isolated and characterized from the sponge *Tedania ignis*, were later found to be produced by associated *Micrococcus* (**Figure 2**) (Stierle et al., 1988).

Due to the overall paucity of information about specific microbially produced metabolites on marine sponges, there is little information on the roles they may play for their sponge hosts. Recently, however, the genomes of three marine sponge associated actinomycetes (*Micromonospora* sp. RV43, *Rubrobacter* sp. RV113, and *Nocardiopsis* sp. RV163) were sequenced, and subsequently mined for the presence of secondary metabolite producing gene clusters (Horn et al., 2015). All three species under investigation contained gene clusters required to produce NP of known antibiotic and antifungal activity. To study how bacterial symbionts affect the ecology of the sponge, Mehbub et al. (2016) performed controlled aquarium experiments designed to test the effects of increased levels of a sponge-associated actinomycete, *Streptomyces* ACT-52A, on the sponge’s microbial community, metabolite profile, and bioactivity. When *Streptomyces* ACT-52A was exogenously added to the water in the aquarium, they observed shifts in the microbial community, accompanied by a change in metabolite profile composition and the bioactivity of sponge extracts. Though it is still not clear if the changes in metabolite production were of microbial or sponge origin, it is clear that interactions between the sponge and its associated bacteria can impact NP biosynthesis and microbial diversity. We can envision this as a system where the effect of mutant actinomycetes, deficient in the production of a specific NP, may be tested. A possible next step would be to investigate how the health and function of the sponge, and its associated microbiome, may impact the marine ecosystem as a whole.

Coral reefs themselves have also been of interest with respect to bioprospecting as they house an immense diversity of secondary metabolites (Leal et al., 2013). A large number of these metabolites, however, are produced by symbiotic bacteria living in the corals (Leal et al., 2013). In one instance, thiocoraline, a thiodepsipeptide, was isolated from a coral associated marine actinomycete, *Micromonospora marina* (Romero et al., 1997). Though thiocoraline was shown to have anti-tumor and antibiotic activity *in vitro*, no *in situ* experiments have been done, thus the ecological impacts of this metabolite on the corals remains unexplored (**Figure 2**). These and other bacterial symbionts potentially have a number of functions with regard to reef health, including protection from invading pathogens. However, the culturing of the majority of marine bacteria remains a major impediment, and thus the analysis of the majority of reef associated actinomycetes remains elusive. Beyond this, large amounts of reef biomass are required for the isolation of coral-associated metabolites, and while the aquaculturing of reefs represents a potentially viable option to produce reef associated metabolites (Leal et al., 2013), the complexity of marine environments is difficult to replicate, and thus the chemical ecology, and symbiotic microbiome, may be drastically different in laboratory experiments (Hay, 1996).

Collectively reefs occupy <0.2% of the world’s oceans (**Figure 2**), yet are estimated to be one of the most biodiverse ecosystems on the planet (Knowlton et al., 2010), that includes a panoply of coral and sponge species. Taken together, the studies described above point to a long lasting symbioses between corals, sponges, and actinomycetes. As for the other systems addressed in this review, assessing the contribution of actinomycete NP to the overall health and stability of reef ecosystems is challenging. However, any advantage conferred by actinomycete symbiosis is likely to have ecosystem-wide implications. Further studies aimed at understanding the role of these compounds in simplified, controlled aquarium settings may provide a window into the chemical underpinnings of these ecosystems.

## Concluding Remarks

Accurately assessing the role of a given bacterial species in its natural environment is a daunting task. However, as we transition to the ‘age of the microbiome,’ questions about microbial community function, and community impact on their respective ecosystems, will become increasingly important. Actinomycetes are interesting to consider in this regard since the role played by their extensive NP repertoires has long been the source of speculation. Beyond this, at a practical level, understanding the roles and ecological drivers that stimulate production of these compounds may open new avenues to NP discovery. Only by conducting these kinds of studies, will we begin to understand how these molecules function in natural settings, and what advantages might be gained for the organisms that make these compounds. We suggest that knowledge about the ecological and chemical contexts in which these molecules are used by the producing organisms may provide insights into how we can minimize the development of pathogen resistance and extend the working life of antibiotics in the clinic.

What does it take to effectively evaluate the impact of a NP *in situ*? The framework for considering this question could be adapted from the fundamental principles of Koch’s postulates. Namely, the NP should be detectable *in situ*, removing the producing organism from the system should eliminate the NP and result in a measureable effect on the system, and replacing the producing organism should lead to detection of the compound *in situ* and reverse the effect of removing it. Undoubtedly, the most rigorous approach would be to replace the producing organism with a mutant defective in its ability to produce the NP in question, followed by verifying the absence of the NP and assessment of the effect on the system overall. These stringent criteria are obviously challenging to implement in practice, especially in natural settings.

Determining the ecological drivers of NP biosynthesis *in situ* may ultimately prove even more challenging than assessing the impact of these compounds. However, we suggest that understanding the effect that a given NP has on the surrounding microbial community may provide valuable context for considering what cues may be connected to its production. Another starting point will be to understand cues that drive actinomycete specialized metabolism in simple, binary interactions. Surprisingly, while it has been shown many times that binary interspecies interactions can lead to activation of NP biosynthesis (Ueda et al., 2000; Yamanaka et al., 2005; Onaka et al., 2011; Vetsigian et al., 2011; Traxler et al., 2013; Kinkel et al., 2014; Abrudan et al., 2015), a molecular/physiological understanding of this phenomenon remains largely elusive. Only in one case, described by Yamanaka et al. (2005), was a molecule identified as triggering antibiotic production in another nearby strain. In that instance, the stimulatory molecule revealed to be the common siderophore desferrioxamine E. Accordingly, understanding the chemical and mechanistic nature of these interactions is an active area of research being undertaken in multiple labs. Beyond binary interactions, simplified synthetic communities that can be experimentally manipulated will also offer a tractable starting point for assessing connections between cues, NP, and ecosystem effects.

However, difficult it may be to carry out studies on complex natural systems that include microbial communities, the development of new tools provides a reason to be optimistic about future efforts aimed at understanding the ecology of NP. Specifically, advances in mass spectrometry imaging and direct chemical sampling of microhabitats via technologies like NanoDESI MS are making detection of these fascinating molecules *in situ* a much more tractable prospect. One predictable outcome of removing a molecule with antibiotic or antifungal activity from a microbial community might be a shift in the overall community composition. Fortunately, the best method for assessing these changes, e.g., metagenomic sequencing, has also become radically more accessible. Moreover, as the study of microbiomes advances with the development of model microbial communities and more portable technologies to enable measurement of key microbiome parameters in the field (Alivisatos et al., 2015; Blaser et al., 2016) the questions posed here will become more tractable.

## Author Contributions

All authors listed, have made substantial, direct and intellectual contribution to the work, and approved it for publication. SB and BB are the primary authors of this manuscript.

## Conflict of Interest Statement

The authors declare that the research was conducted in the absence of any commercial or financial relationships that could be construed as a potential conflict of interest.
